# The cell-type specific uptake of polymer-coated or micelle-embedded QDs and SPIOs does not provoke an acute pro-inflammatory response in the liver

**DOI:** 10.3762/bjnano.5.155

**Published:** 2014-09-02

**Authors:** Markus Heine, Alexander Bartelt, Oliver T Bruns, Denise Bargheer, Artur Giemsa, Barbara Freund, Ludger Scheja, Christian Waurisch, Alexander Eychmüller, Rudolph Reimer, Horst Weller, Peter Nielsen, Joerg Heeren

**Affiliations:** 1 Department of Biochemistry and Molecular Cell Biology, University Medical Center Hamburg-Eppendorf; Martinistrasse 52, 20246 Hamburg, Germany; 2 Department of Genetics and Complex Disease, Harvard School of Public Health, 665 Huntington Avenue, Boston, 02115 MA, USA; 3 Department of Chemistry, Massachusetts Institute of Technology, 77 Massachusetts Ave., Cambridge, MA 02139, USA; 4 Institute of Physical Chemistry and Electrochemistry, Technical University of Dresden, 01062 Dresden, Germany; 5 Department of Electron Microscopy and Micro Technology, Heinrich-Pette Institute, Martinistrasse 52, 20246 Hamburg, Germany; 6 Institute of Physical Chemistry, University of Hamburg, Grindelallee 117, 20146 Hamburg, Germany

**Keywords:** hepatocytes, inflammation, Kupffer cells, liver sinusoidal endothelial cells, nanoparticle toxicity, nanoparticle uptake, quantum dots, superparamagnetic iron-oxide nanocrystals

## Abstract

Semiconductor quantum dots (QD) and superparamagnetic iron oxide nanocrystals (SPIO) have exceptional physical properties that are well suited for biomedical applications in vitro and in vivo. For future applications, the direct injection of nanocrystals for imaging and therapy represents an important entry route into the human body. Therefore, it is crucial to investigate biological responses of the body to nanocrystals to avoid harmful side effects. In recent years, we established a system to embed nanocrystals with a hydrophobic oleic acid shell either by lipid micelles or by the amphiphilic polymer poly(maleic anhydride-*alt*-1-octadecene) (PMAOD). The goal of the current study is to investigate the uptake processes as well as pro-inflammatory responses in the liver after the injection of these encapsulated nanocrystals. By immunofluorescence and electron microscopy studies using wild type mice, we show that 30 min after injection polymer-coated nanocrystals are primarily taken up by liver sinusoidal endothelial cells. In contrast, by using wild type, *Ldlr*^-/-^ as well as *Apoe*^-/-^ mice we show that nanocrystals embedded within lipid micelles are internalized by Kupffer cells and, in a process that is dependent on the LDL receptor and apolipoprotein E, by hepatocytes. Gene expression analysis of pro-inflammatory markers such as tumor necrosis factor alpha (TNFα) or chemokine (C-X-C motif) ligand 10 (Cxcl10) indicated that 48 h after injection internalized nanocrystals did not provoke pro-inflammatory pathways. In conclusion, internalized nanocrystals at least in mouse liver cells, namely endothelial cells, Kupffer cells and hepatocytes are at least not acutely associated with potential adverse side effects, underlining their potential for biomedical applications.

## Introduction

The superior optical properties of QDs compared to organic dyes render them promising candidates for the demands of sophisticated in vivo imaging in biomedical diagnosis [1]. QDs have been used for fluorescence-based imaging by several investigators, however, the chemical composition of their inorganic crystal core, e.g., cadmium, raised concerns about their biocompatibility [2]. Thus, it is not surprising that studies employing various cell culture systems described toxic effects of QDs [3–4]. Iron-containing superparamagnetic iron oxide nanocrystals (SPIOs) used for magnetic resonance imaging (MRI) have a relative good reputation given that iron is an essential trace element and it can be assumed that iron from degraded SPIOs is transferred to the body iron stores. Nevertheless, iron-induced acute toxic reactions, probably related to the generation of reactive oxygen species, have been described in vitro after uptake of large amount of various SPIOs [5]. However, cell culture studies like the ones described above disregard the complexity of the physiological system that is exposed to nanocrystals. Cellular distribution, organ-specific metabolism, cell–cell interaction and the activation of professional cells of the innate and adaptive immune system are likely to influence the biological response of nanocrystals-loaded, parenchymal cells. Thus, there is need for in vivo studies addressing the biological fate of QDs as well as SPIOs with regard to potential harmful effects in whole organisms.

Recent research investigating the metabolism and excretion of nanocrystals focuses on exposure routes. The excretion of injected nanocrystals was found to depend strongly on the size and to some extent on the surface. For direct renal excretion the upper size limit in the series was a hydrodynamic diameter of 5.6 nm [6]. Larger nanocrystals apparently remain within the circulation before they are taken up by macrophages of the mononuclear phagocytic system (MPS) [7]. Consequently, larger nanocrystals are exposed to cellular degradation mechanisms of macrophages, e.g., the acidic environment of lysosomes and even more harsh conditions in phagosomes containing also hydrogen peroxide [8]. This might disrupt the surface coating and dissolve ions out of the inorganic crystal core. Such a degradation process will alter or diminish the optical properties of QDs and expose the cells and subsequently the whole body to toxic metal ions. Recently, the group of Bruchez described the changes in the optical properties of injected QDs in mice. They found that QDs persist in lymph nodes over a period of two years. These nanocrystals retain fluorescence but with a blue shifted emission, which is suggestive for a release of some cadmium ions from the inorganic core [9]. Interestingly, even after two years no obvious signs of cadmium-induced toxicity were observed. Other studies focus on the quantification of cadmium as tracer for injected nanocrystals. The laboratory of Lin investigated the biodistribution of QDs in mice over four months [10]. In this study, a slow redistribution of the chemical components from peripheral organs to the kidneys was observed. These findings are supported by a study in rats describing a time-dependent increase in the cadmium concentration over 30 days after injection of QDs in the kidneys indicating that these nanocrystals are slowly degraded in vivo [11]. In summary, despite the breakdown of cadmium-containing nanocrystals, pathological alterations in response to the injection of CdSe–ZnS core–shell QDs were observed only in some of these studies [9,11], while others found ultrastructural changes in the kidneys [10] or the spleen [12]. These results are surprising taking into account that the metals within the inorganic core are potentially toxic. Apparently, QDs with a special nano-sized formulation and surface passivation prevent acute toxic effects. Given that only some of the reports found pathological effects in a clinical sense, parameters that are more sensitive such as inflammatory markers or changes in metabolite levels should be determined to access the biological response to nanocrystals in vivo. This is even more important as plasma proteins rapidly bind to the surface of nanoparticles to form a protein corona that influences distinct pathophysiological effects such as haemolysis or nanoparticle uptake [13–14].

In most studies so far, complex surface modification was carried out to achieve water-solubility of hydrophobic QDs or SPIOs [15–16]. Another way to make nanocrystals hydrophilic is the embedding of QDs or SPIOs into the core of lipid micelles [17–19]. After injection, these nanocrystal-containing lipid micelles are internalized by adipose tissues and predominantly by the liver [18,20]. The liver is not only the most important organ for metabolism and detoxification but also the major target organ for injected polymer-coated QDs and SPIOs [21]. However, little is known about the contribution of different cell types involved in nanocrystals uptake as well as biological responses to nanocrystals with regard to hepatic cell types.

Here we demonstrate that polymer coat-embedded CdSe/CdS/ZnS-based QDs and SPIOs are internalized primarily by liver sinusoidal endothelial cells. In contrast, nanocrystals transported by lipid micelles are detected within hepatic macrophages, the Kupffer cells, and within liver parenchymal cells, the hepatocytes. Intriguingly, even 48 h after injection, neither changing the embedding procedure nor the cellular targeting provoked any pro-inflammatory reaction in response to the uptake of QDs or SPIOs in vivo.

## Results and Discussion

**Figure 1:**
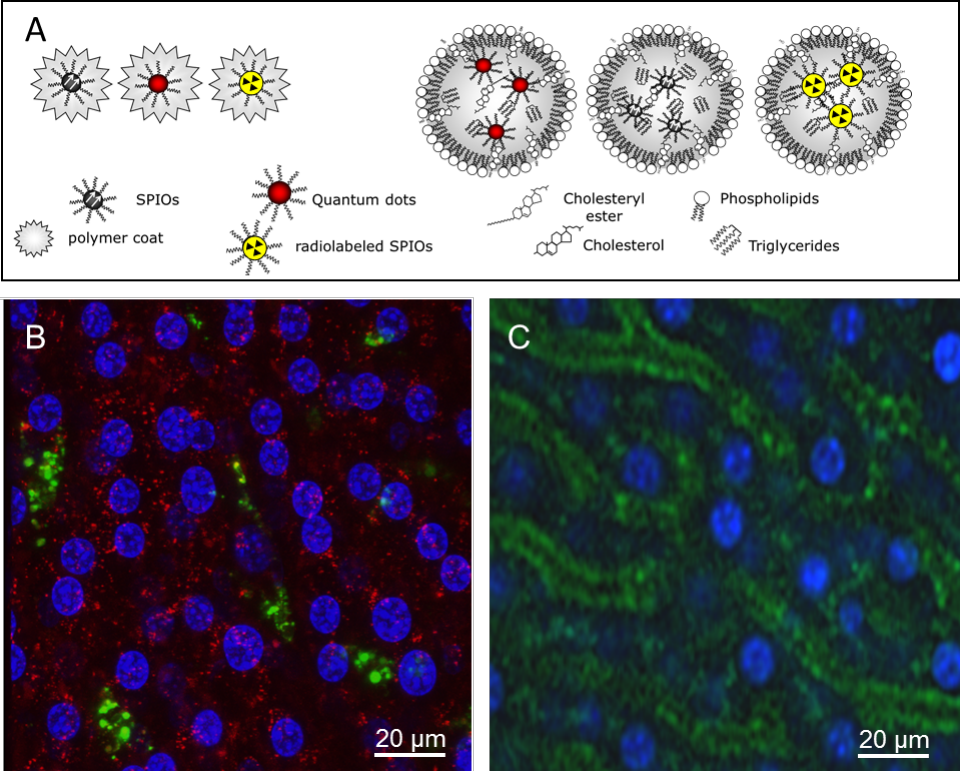
Characterization of nanocrystals and uptake into liver cells in vivo. (A) Oleic acid-stabilized SPIOs, radiolabeled SPIOs (59Fe-SPIOs) or QDs are embedded in PMAOD-polymer or in lipid micelles as indicated in the model. (B) Native DID-labeled LDL (red) and QDs-micelles (green) or (C) polymer-embedded QDs (green) were injected into wild type C57BL/6 mice via a tail vein catheter. Nuclei were stained by intraperitoneal injection of the fluorescence dye Hoechst 33342. 30 min after injection, the liver was excised and directly placed on the confocal microscope. As shown in (B), LDL are internalized by hepatocytes (red) whereas signals from QDs embedded within lipid micelles are found associated with hepatic Kupffer cells (green). (C) shows that polymer-embedded QDs are primarily associated with endothelial cells lining the sinusoids of the liver. Scale bars = 20 µm.

In order to investigate potential pro-inflammatory pathways of injected nanocrystals and also to study their hepatocellular route, we recently established the methodology for the embedding of QDs, SPIOs and ^59^Fe-SPIOs either by an amphiphilic polymer coat [21] or by the incorporation into the lipid core of micelles [17–18], as indicated in the schematic model (Figure 1A).

**Figure 2:**
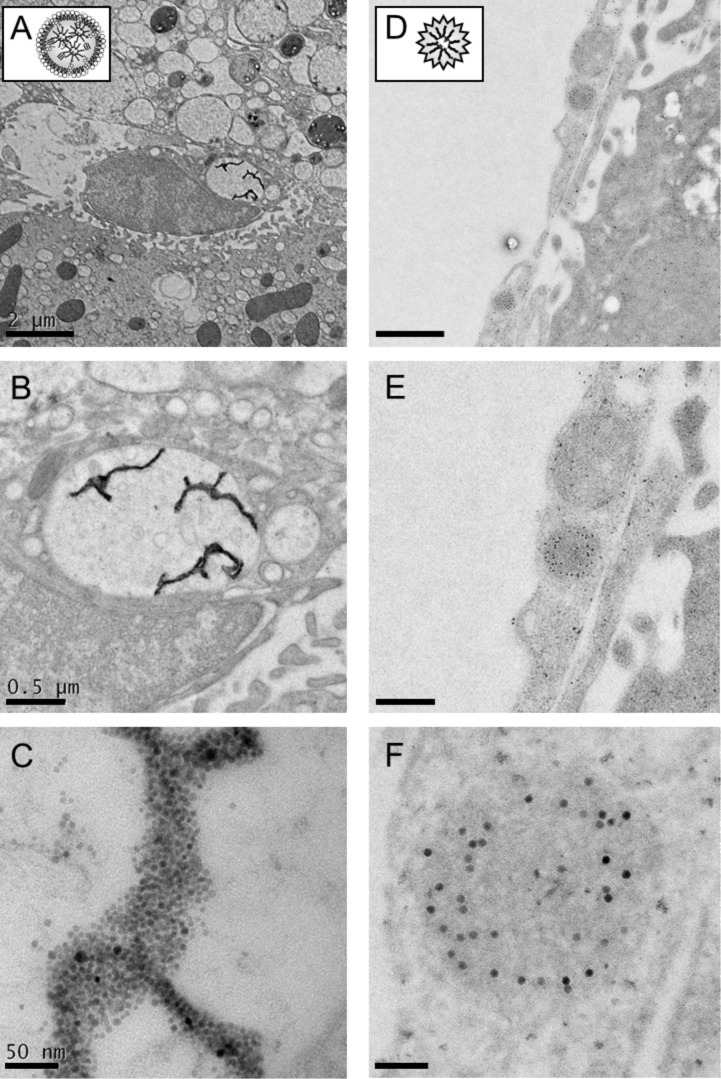
Cryo-electron microscopy of hepatic nanocrystals uptake. SPIOs-micelles (left panel) or polymer-embedded SPIOs (right panel) were injected into wild type C57BL/6 mice. 30 min after injection, mice were perfused with 2% paraformaldehyde in PBS and livers were processed for electron microscopy. (A–C) The pictures highlight a Kupffer cell 30 min after the injection of SPIOs-labelled lipid micelles. Clustered nanocrystals of lipid micelles can be detected within intracellular compartments, probably a lipid droplet-like structure of the cell. (D–F) Polymer-coated SPIOs can be found in endosomal structures of endothelial cells. Scale bars correspond to 2 µm (A,D), 0.5 µm (B,E) and 50 nm (C,F), respectively.

The liver rapidly clears polymer-coated ^59^Fe-SPIOs [19], however the exact molecular mechanisms and cell types involved in the processing of these particles are not clarified in detail. Similar to lipoproteins, intravenously injected QDs– or SPIOs–lipid micelles reach the systemic circulation and are immediately converted by the hydrolytic activity of the enzyme called lipoprotein lipase [22–23], which is located at the luminal site of endothelial cells in adipose tissue and muscles. By using nanocrystals-based imaging technology we recently could show that entire lipid micelles are internalized by activated brown adipose tissue, the organ responsible for heat production in order to defend the body against cold [20,24]. During peripheral processing within adipose tissues, the remaining lipid micelles become enriched with apolipoprotein E within the vascular system. These particles are then taken up primarily by hepatocytes in a process that is dependent on hepatic lipoprotein receptors such as the LDL receptor and its ligand apolipoprotein E, indicating that the nanocrystals did not influence the specificity of the metabolic process [18]. Here we show that substantial amounts of injected QDs–lipid micelles were not only internalized by hepatocytes but also targeted to non-parenchymal hepatic cells, most likely Kupffer cells (Figure 1B, green). Concomitantly injected native LDL were detected only within parenchymal hepatocytes (Figure 1B, red). In contrast, polymer-coated nanocrystals selectively accumulated in liver sinusoidal endothelium (Figure 1C, green). These data indicate that associated apolipoproteins and lipid moieties of lipid micelles as well as PMAOD of the polymer coat most likely determine the cell type-specific uptake of nanocrystals. In order to investigate subcellular targeting of internalized nanocrystals, we performed cryo-electron microscopy after the injection of either SPIOs–lipid micelles (Figure 2A–C) or polymer-coated SPIOs (Figure 2D,E). These studies confirmed Kupffer cell targeting of nanocrystals transported by lipid micelles (Figure 2A). Higher magnification revealed the subcellular transport and storage of numerous SPIOs within lipid droplet-like structures (Figure 2B,C) suggesting that after their internalization nanocrystals are not degraded within 30 min. The storage of oleic acid-coated nanocrystals within these lipophilic, intracellular compartments probably inhibits the release of free iron ions thereby preventing the generation of reactive oxygen species and inflammatory responses in Kupffer cells [25–26]. Figure 2D,E demonstrated that polymer-coated SPIOs are primarily detected within endosomal compartments of liver sinusoidal endothelial cells (LSEC). This specialized cell type known to effectively internalize small, negatively charged particles and gut-derived molecules [27–28] can induce a tolerance to internalized gut-derived substances and usually does not support pro-inflammatory T cell effector responses [28–30]. Thus, it is quite unlikely that nanocrystals internalized by LSEC provoke an acute pro-inflammatory response.

**Figure 3:**
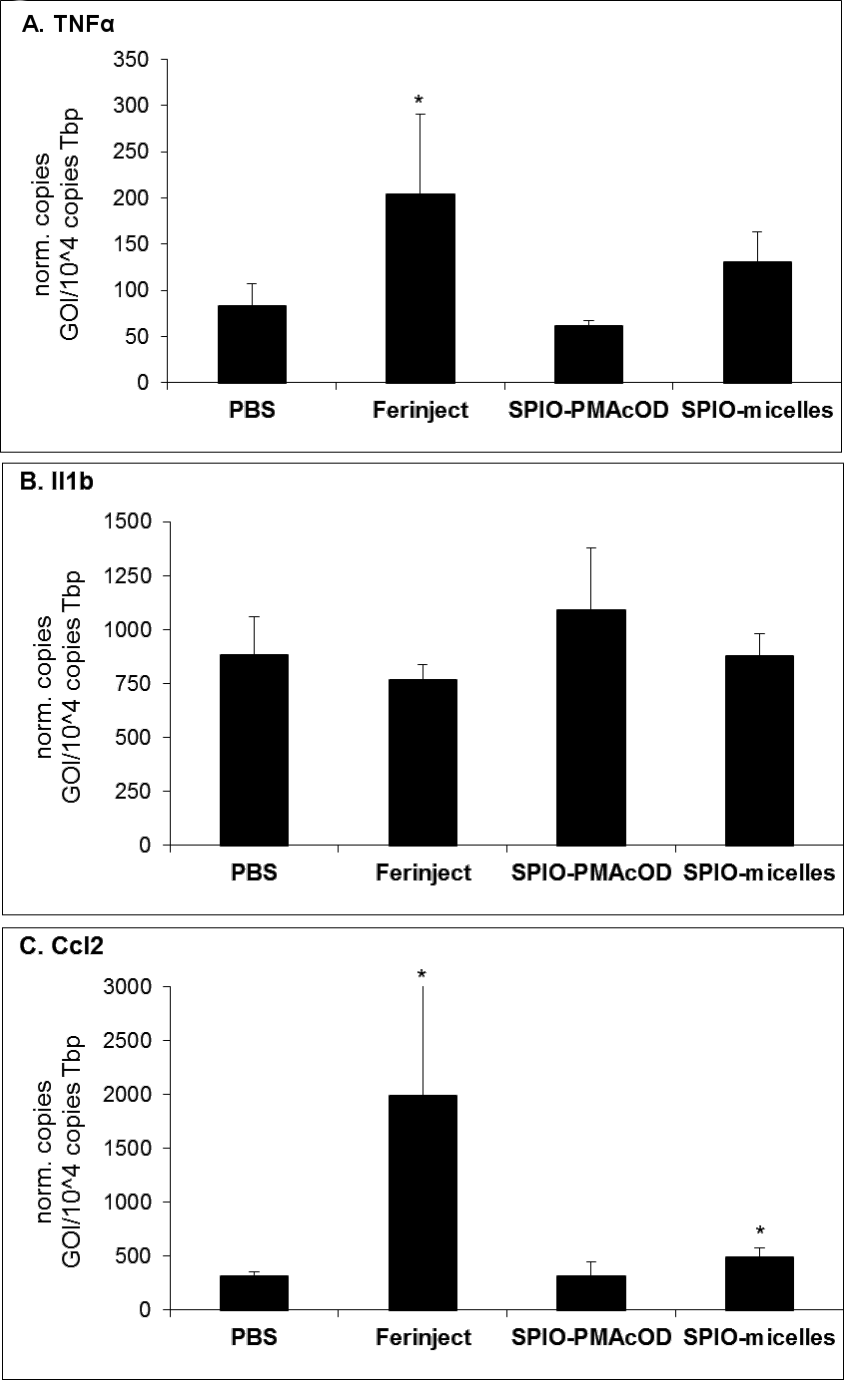
Impact of internalized SPIOs on gene expression. Wild type BALB/c mice were intravenously injected with PBS (iron free control), Ferinject^®^ (4.6 mg iron per mouse); polymer-coated SPIOs (100 µg iron per mouse) and SPIOs-micelles (50 µg iron per mouse). Gene expression analysis was performed for (A) TNFα, (B) Il1b and (C) Ccl2. The injection of high iron doses by using Ferinject^®^ but not the injection of SPIOs increased the expression of pro-inflammatory TNFα and Ccl2. Mean values +/− s.d. with *n* ≥ 4, *p < 0.05

In order to test this hypothesis, we measured pro-inflammatory markers by gene expression analysis after the injection of SPIO nanocrystals embedded by different coats. Intravenous injection of Ferinject^®^ (contains ferric carboxymaltose that is commonly given to treat iron deficits) at high doses caused a significant induction of pro-inflammatory markers such as TNFα and Ccl2 (Figure 3A,C). In contrast, polymer-coated SPIOs had no effect whereas SPIOs embedded within lipid micelles had a modest effect on the expression of these pro-inflammatory genes (Figure 3A,C). The expression of Il1b is regulated in a feed-forward process by an intracellular multiprotein oligomer, the so-called-inflammasome [31], which can be activated by intracellular aggregates such as ureate or cholesterol crystals [32]. Notably, hepatic internalization of Ferinject^®^ or SPIOs delivered by polymer or lipid micelles did not influence Il1b gene expression underlining the non-inflammatory properties of SPIOs independent of the hepatic cell type responsible for the uptake.

**Figure 4:**
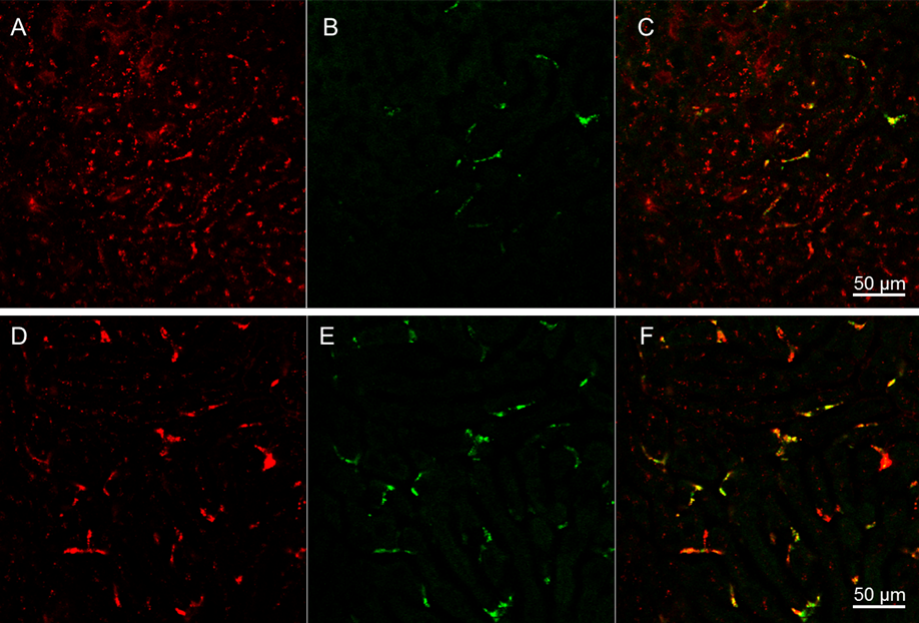
LDL receptor dependent uptake of QDs-micelles and LDL. Native LDL (red) and QDs-micelles (green) were injected into wild type C57BL/6 mice (upper panel) or into LDL receptor deficient mice (lower panel). 30 min after injection, the livers were excised and directly placed on the microscope for confocal imaging. In the wild type situation, LDL particles were predominantly internalized by hepatocytes (A) whereas.

To further delineate molecular mechanisms involved in processing and interaction of nanocrystals with hepatocytes and Kupffer cells, we visualized the uptake of both human LDL and QDs–lipid micelles in wild type mice (Figure 4, upper panel) or in transgenic mice lacking the LDL receptor (Figure 4, lower panel). After injection, native human LDL were internalized by wild type hepatocytes (Figure 4A) whereas the bulk of QDs were primarily detected within star-shaped cells, most likely Kupffer cells (Figure 4B). The yellow colour in the merged image (Figure 4C) indicated that minor amounts of native LDL are also internalized by non-parenchymal Kupffer cells (Figure 4C). In the absence of the LDL receptor, both LDL (Figure 4D,) and QDs-micelles (Figure 4E) were detected within Kupffer cells as shown by the yellow fluorescence in the merged image (Figure 4F). These data demonstrate an alternative LDL and lipid-micelles uptake pathway mediated by Kupffer cells independently of the LDL receptor pathway.

QDs-micelles were found within star-shaped Kupffer cells (B). The yellow colour in the merged image indicates that LDL particles are also detectable within Kupffer cells (C). In LDL receptor deficient mice, LDL (D) and QDs-micelles (E) were found in star-shaped Kupffer cells as indicated by the yellow colour of the merged image (F). Scale bars = 50 µm.

**Figure 5:**
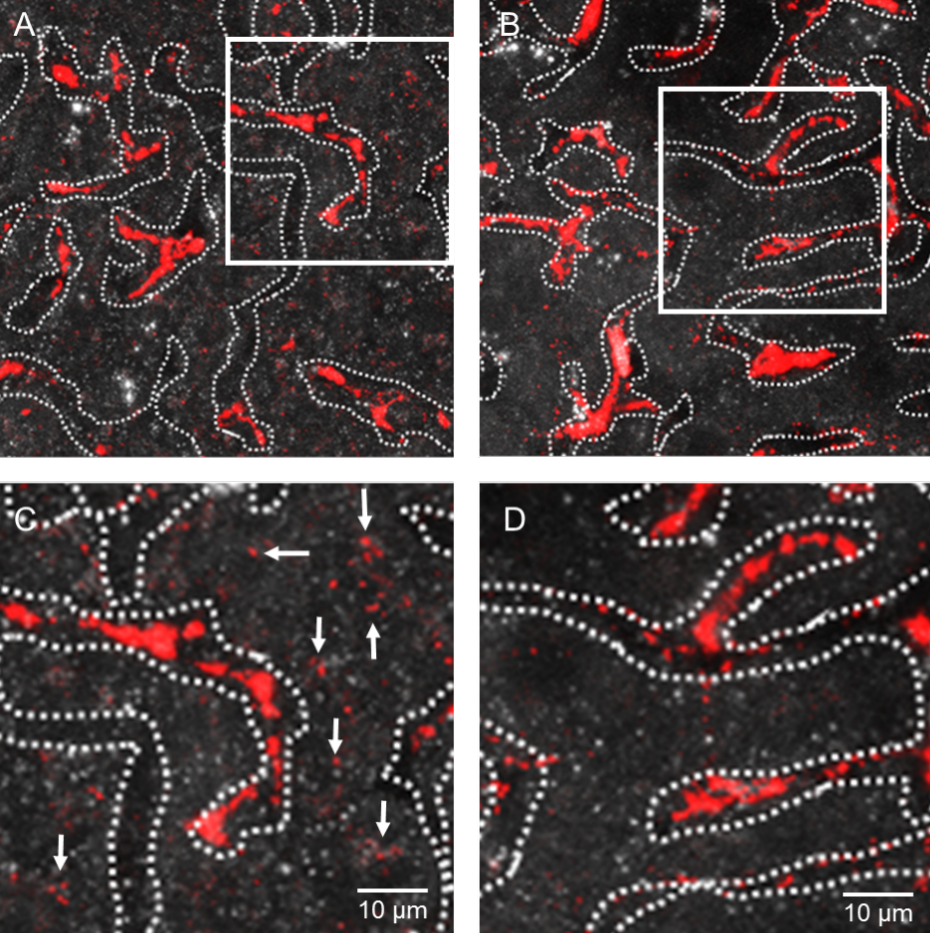
QDs-micelles uptake into hepatocytes is dependent on apolipoprotein E. QDs-micelles (red) were injected into wild type mice (left panel) or into apolipoprotein E-deficient mice (right panel). 30 min after injection, livers were excised and directly analysed by using confocal imaging. Liver sinusoids were visualized by the reflection mode in the unstained tissue and the capillary lumen is surrounded by dashed lines. In the wild type situation, QDs-derived fluorescence were found in Kupffer cells, which are located within the lumen of liver sinusoids (A). In addition, high magnification of the highlighted square revealed uptake of QDs-micelles into hepatocytes (C, indicated by the arrows). In apolipoprotein E-deficient mice, QDs can mainly be detected within the lumen of liver sinusoids (B,D), which indicates that the QDs-micelle uptake into hepatocytes is dependent on apolipoprotein E.

Next we investigated the role of apolipoprotein E for the uptake of QDs-micelles into liver cells (Figure 5). In order to visualize sinusoids of the unfixed liver, we performed confocal microscopy using the reflection mode and marked the liver sinusoids by dashed lines. In wild type mice, QDs-micelles (red) were detected with star-shaped cells suggesting an uptake into Kupffer cells, which are located within the lumen of liver sinusoids (Figure 5A). At higher magnification (Figure 5C), a punctate pattern of QDs-derived fluorescence can be detected in cells that are located next to liver sinusoids suggesting that substantial amounts of QDs-micelles are transported into endosomal compartments of wild type hepatocytes (see arrows in Figure 5C). In apolipoprotein E deficient mice, QDs-micelles are mainly detected, even at high magnification, in cellular structures located within liver sinusoids (Figure 5B,D) suggesting an impaired uptake by canonical lipoprotein receptor-mediated apolipoprotein E-dependent endocytosis into hepatocytes. Future studies by using radiolabelled QDs are needed to quantify the precise contribution of different hepatic cell types for QDs-micelles uptake. However, only 5–10% of liver cells are Kupffer cells [25,33] and therefore it is implausible that this cell type is quantitatively important for lipid micelles internalization. Nevertheless, these specialized macrophages bearing high phagocytic activity are part of the innate immune system [25] and their stimulation can activate the transcription of pro-inflammatory factors such as TNFα, a cytokine, provoking collagen synthesis and fibrosis [34].

**Figure 6:**
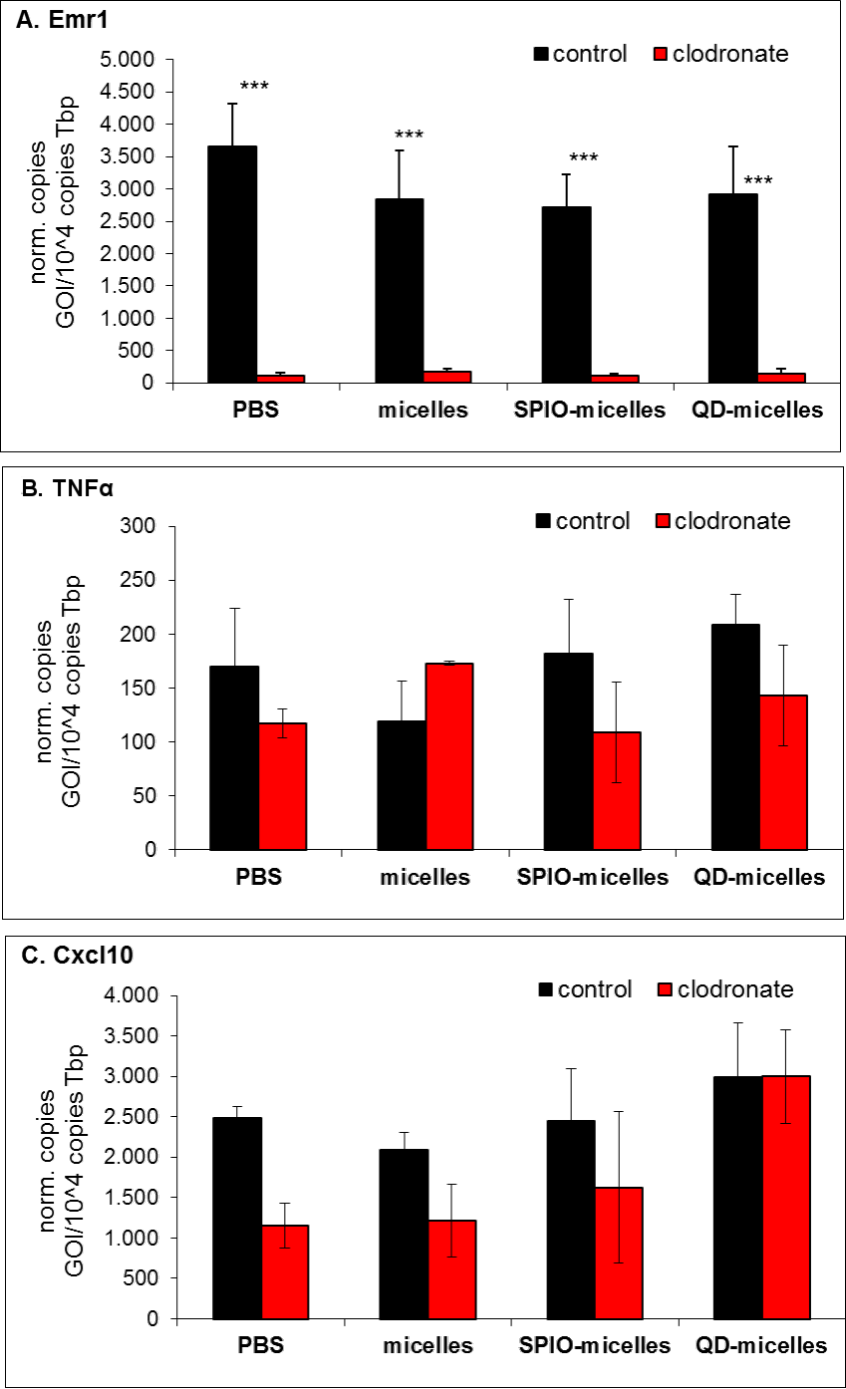
Impact of QDs– and SPIOs–lipid micelles on hepatic gene expression after ablation of Kupffer cells. Wild type und clodronate-treated BALB/c wild type mice were intravenously injected with PBS (control), lipid micelles, SPIOs-micelles and QDs-micelles. Livers were harvested 4 h after injection and gene expression analysis was performed for (A) Emr1, (B) TNFα and (C) Cxcl10. Mean values +/− s.d. with *n* ≥ 4, ***p < 0.001.

To clarify the quantitative role of Kupffer cells for potential harmful effects of injected nanocrystals, clodronate containing liposomes were injected to ablate Kupffer cell populations in the liver selectively [35]. The F4/80 (encoded by Emr1) molecule is solely expressed on the surface of macrophages and serves as a marker for mature macrophage tissues, including Kupffer cells in liver, splenic red pulp macrophages or brain microglia [36]. Two days after clodronate treatment, Emr1 expression was undetectable demonstrating effective ablation of Kupffer cells in our system (Figure 6A). Furthermore, consecutive injections of pure lipid micelles or micelles containing either QDs or SPIOs in the presence or absence of Kupffer cells had no acute influence on TNFα or Cxcl10 expression (Figure 6B, C) indicating that heavy metals such as cadmium released from QDs or iron released from SPIOs did not acutely influence the inflammatory status of the liver.

However, specific target cells can be of transient relevance and heavy metals released after QDs or SPIOs uptake may traverse through different target cell types in a time-dependent manner. Given the limitation of the study that gene expression of pro-inflammatory markers was analysed 48 h (Figure 3) or 4 h (Figure 6) after the injection of nanoparticles, we cannot exclude that different target cells in different organs such as the kidney, spleen, adipose tissues or the bone may be relevant at time points other than the ones we investigated in the current study.

## Experimental

### Animals and diets

All experimental procedures were performed with approval from the animal care committees responsible for the University Medical Center Hamburg-Eppendorf. Animals were housed at 22 °C with ad libitum access to standard laboratory chow diet and water. We used male age-matched (10–12 weeks) wild type mice (BALB/c or C57BL/6J) or *Ldlr*^-/-^ as well as *Apoe*^-/-^ mice (obtained from The Jackson Laboratory) which were fasted 4 h prior to the experiment.

### Preparation of polymer-coated nanocrystals

Encapsulation of nanocrystals was achieved as described [37] with slight modifications: 2 mL poly(maleic anhydride-*alt*-1-octadecene) (PMAOD) solution (concentration: 0.01 g/mL in CHCl_3_) were added to a solution of either 2 mg oleic acid stabilized SPIO, QDs or ^59^Fe-SPIOs [21] dissolved in 2 mL. The solvent was evaporated by N_2_, and solution was sonicated in 2 mL TBS buffer. Afterwards, the solution was heated to 60 °C and aggregates were removed by centrifugation. An excess of polymer was removed by ultracentrifugation (1 h, 50,000*g*, 4 °C). Finally, the solution was filtered sequentially through a 0.45, 0.2, and 0.1 μm Millipore filter. Based on dynamic light scattering (DLS) measurements, the size of polymer-coated nanocrystals is 25 nm. These polymer-coated nanoparticles are negatively charged due to the formation of carboxyl groups at the surface.

### Labelling of lipid micelles with nanocrystals and fluorescent lipid tracers

Lipids derived from isolated human lipoproteins were extracted by the method of Folch. A detailed method for the labelling of triglyceride-rich lipoproteins (TRL) with nanocrystals was described recently [18]. Briefly, for embedding 10 mg of the lipid extract was dissolved in chloroform and mixed with either SPIOs, QDs or with ^59^Fe-SPIOs [18,20]. After the solvent was evaporated, 1 mL of PBS was added and nanocrystals-containing lipid micelles were formed by sonication. Potential aggregates were removed by filtration using a 0.45 µm membranous filter prior to intravenous injection. As determined by DLS measurements, the sizes of QDs- or SPIOs-labelled lipid micelles are approximately 250 nm. After intravenous injection, lipid micelles are rapidly hydrolyzed to particles smaller than 100 nm in vivo [38]. Based on agarose gel electrophoresis, the surface charge of the QDs–lipid micelles is negative. To produce native human LDL labelled with the red dye DiD (1,1'-dioctadecyl-3,3,3',3'-tetramethylindodicarbocyanine perchlorate, Invitrogen), we incubated 5 mg LDL over night with DiD at 37 °C by gentle shaking. To remove free dye, QDs–lipid micelles or DiD–LDL were isolated by gel chromatography using a PD-10 column (GE Healthcare). We injected 200 µL per mouse of QDs–lipid micelles (lipid concentration of QDs-lipid micelles stock solution was 10 mg/mL) or 200 µL of DiD–LDL per mouse (protein concentration of DiD–LDL was 2 mg/mL, total lipid concentration of DiD–LDL was 8 mg/mL).

### Confocal and electron microscopy

For intravital microscopy, livers of anaesthetized mice were externalized and nanocrystals uptake was visualized by using a confocal microscope equipped with a resonant scanner (Nikon A1R). In order to visualize nuclei, 100 µL of the fluorescence dye Hoechst 33342 (0.2 mg/mL) per mouse was injected intraperitoneally. QDs- or DiD-labelled probes were injected via a tail vein catheter. For cryo electron microscopy, polymer- or lipid micelles-coated SPIOs-were intravenously injected into wild-type mice. 30 min after injection, mice were sacrificed, liver biopsies were taken and processed for transmission electron microscopy (TEM) as described [18,20]. Micrographs were obtained with a FEI Eagle 4k CCD camera and a Technai 20 TEM operated at 200 kV.

### Clodronate-mediated ablation of Kupffer cells

Procedure was performed as described recently [35]. Briefly, mice were injected intravenously in the tail vein with 200 µL Clodronate liposome solution (ClodronateLiposomes.org, Amsterdam, Netherland) or empty liposomes as control two days prior to the experiments.

### Gene expression analysis

Gene expression analysis was performed as described [39]. Briefly, total RNA was isolated and cDNA was prepared according to the manufacturer’s instructions (Applied Biosystems). Real-time quantitative RT-PCR was performed by using assay-on-demand primer/probe sets supplied by Applied Biosystems (assay IDs are available upon request). Relative expression was calculated by normalization to selected housekeeper mRNA (TATA-binding protein: *Tbp*) by ΔΔCt method. Data are reported as copy number relative to housekeeper.

### Statistics

To assess statistical significance a two-tailed, unpaired Student’s *t*-test was performed. A value of *p* < 0.05 was considered significant.
